# Deletion of the low-molecular-weight glutenin subunit allele *Glu-A3a* of wheat (*Triticum aestivum* L.) significantly reduces dough strength and breadmaking quality

**DOI:** 10.1186/s12870-014-0367-3

**Published:** 2014-12-19

**Authors:** Shoumin Zhen, Caixia Han, Chaoying Ma, Aiqin Gu, Ming Zhang, Xixi Shen, Xiaohui Li, Yueming Yan

**Affiliations:** Laboratory of Molecular Genetics and Proteomics, College of Life Science, Capital Normal University, 100048 Beijing, China

**Keywords:** Wheat, *Glu-A3a*, Molecular cloning, Dough strength, Breadmaking quality

## Abstract

**Background:**

Low-molecular-weight glutenin subunits (LMW-GS), encoded by *Glu-3* complex loci in hexaploid wheat, play important roles in the processing quality of wheat flour. To date, the molecular characteristics and effects on dough quality of individual *Glu-3* alleles and their encoding proteins have been poorly studied. We used a *Glu-A3* deletion line of the Chinese Spring (CS-n) wheat variety to conduct the first comprehensive study on the molecular characteristics and functional properties of the LMW-GS allele *Glu-A3a*.

**Results:**

The *Glu-A3a* allele at the *Glu-A3* locus in CS and its deletion in CS-n were identified and characterized by proteome and molecular marker methods. The deletion of *Glu-A3a* had no significant influence on plant morphological and yield traits, but significantly reduced the dough strength and breadmaking quality compared to CS. The complete sequence of the *Glu-A3a* allele was cloned and characterized, which was found to encode a B-subunit with longer repetitive domains and an increased number of α-helices. The *Glu-A3a*-encoded B-subunit showed a higher expression level and accumulation rate during grain development. These characteristics of the *Glu-A3a* allele could contribute to achieving superior gluten quality and demonstrate its potential application to wheat quality improvement. Furthermore, an allele-specific polymerase chain reaction (AS-PCR) marker for the *Glu-A3a* allele was developed and validated using different bread wheat cultivars, including near-isogenic lines (NILs) and recombinant inbred lines (RILs), which could be used as an effective molecular marker for gluten quality improvement through marker-assisted selection.

**Conclusions:**

This work demonstrated that the LMW-GS allele *Glu-A3a* encodes a specific LMW-i type B-subunit that significantly affects wheat dough strength and breadmaking quality. The *Glu-A3a*-encoded B-subunit has a long repetitive domain and more α-helix structures as well as a higher expression level and accumulation rate during grain development, which could facilitate the formation of wheat with a stronger dough structure and superior breadmaking quality.

**Electronic supplementary material:**

The online version of this article (doi:10.1186/s12870-014-0367-3) contains supplementary material, which is available to authorized users.

## Background

Wheat (*Triticum aestivum* L., 2n = 6x = 42, AABBDD), as a complex allohexaploid species, is one of the most important crops widely cultivated across the world. Wheat grains contain about 10–15% proteins, and are one of the richest protein sources in the human diet. It is well known that wheat breadmaking quality is largely determined by the seed storage proteins present in the grain endosperm, which mainly consist of polymeric glutenins and monomeric gliadins [1,2]. The polymeric glutenins are further subdivided into high-molecular weight glutenin subunits (HMW-GS) and low-molecular-weight glutenin subunits (LMW-GS) according to their mobilities on a sodium dodecyl sulfate-polyacrylamide gel electrophoresis (SDS-PAGE) gel, which determine their dough elasticity, viscosity, and strength [2,3].

LMW-GS can be separated into three groups, the B, C, and D subunits, based on their electrophoretic mobilities on an SDS-PAGE gel. Genetic analysis showed that these subunits are encoded by the *Glu-A3*, *Glu-B3,* and *Glu-D3* loci on the short arms of the chromosomes 1A, 1B, and 1D, respectively [4,5]. Some components were also found to be encoded by genes on the short arms of the group 6 and 7D chromosomes [6]. Based on their N-terminal amino acid sequences, LMW-GS are classified into three subclasses, LMW-m, LMW-s, and LMW-i types, according to the first amino acid residue of the mature protein: methionine, serine, and isoleucine, respectively [6]. The LMW-s type subunit seems to be predominant [7,8]. Typically, the N-terminal amino acid sequence is SHIPGL- in LMW-s type subunits, while LMW-m type subunits have various N-terminal sequences such as METSHIGPL-, METSRIPGL-, and METSCIPGL- [9-11]. The LMW-i type subunit, first reported by Pitts et al. [12], lacks the N-terminal domain and starts directly with the repetitive region of ISQQQQ- after the signal peptide. Although the typical N-terminal domain is absent, LMW-i type subunits can be expressed normally, similar to LMW-m and LMW-s, in the wheat endosperm [13,14]. Most LMW-GSs possess eight cysteine residues, although their positions vary in the different types of subunits, which plays important roles in the formation of intra- and inter-molecular disulfide bonds in the gluten macropolymer [14].

Compared to the *Glu-1* loci encoding HMW-GS, *Glu-3* loci exhibit more extensive allelic variations that are closely related to gluten quality. Early work by Gupta and Shepherd [15] identified and named six alleles at *Glu-A3,* nine alleles at *Glu-B3*, and five alleles at *Glu-D3* loci in common wheat. Recently, 14 unique LMW-GS genes in the wheat cultivar Xiaoyan 54 were identified, four of which were located at *Glu-A3*, three at *Glu-B3*, and seven at *Glu-D3*, based on bacterial artificial chromosome (BAC) library screening and proteomics analysis [16]. The results from a set of Aroona LMW-GS near isogenic lines (NILs) showed that the *Glu-A3* locus has two m-type and 2–4 i-type genes [17]. Analysis of the micro-core collections (MCC) of Chinese wheat germplasm identified more than 15 LMW-GS genes from individual MCC accessions, 4–6 of which were located at the *Glu-A3* locus [18].

Since extensive allelic variations are present at *Glu-3* loci, it is generally difficult to accurately determine the functional properties of individual alleles in different genotypes. To date, the main method used to investigate the effects of different *Glu-3* alleles on dough quality has involved determination of their effects and ranks in NILs. Earlier research on the durum wheat NILs Lira 42 and Lira 45 showed that the LMW-2 type subunit in Lira 45 had significantly greater beneficial effects on gluten strength and breadmaking quality than the LMW-1 subunit in Lira 42 [19]. In bread wheat, *Glu-A3d* possesses three active LMW-GS genes and produces the highest Zeleny sedimentation value (ZSV) and Extensograph maximum resistance (R_max_) [17]. Other reports also showed that the *Glu-A3d* allele had a superior effect on dough strength [20-22]. Recent work on a set of Aroona NILs showed that *Glu-A3b* contributed to a longer midline peak time (MPT) and better raw white Chinese noodle (RWCN) color [23]. Despite the large number of studies performed on the functions of *Glu-3* alleles, more comprehensive and in-depth analyses on the structures and functions of the individual alleles at *Glu-3* loci are still lacking.

In the current work, we conducted the first comprehensive investigation on the molecular characteristics and functional properties of the LMW-GS allele *Glu-A3a* by using a *Glu-A3* deletion line in the Chinese Spring (CS) wheat cultivar in combination with various proteomics and molecular biology approaches. Our results demonstrate that the deletion of *Glu-A3a* significantly reduces wheat dough strength and breadmaking quality. In addition, we demonstrated that *Glu-A3a* results in a longer repetitive domain and more α-helices in the encoded subunit, as well as a higher expression level and accumulation rate during grain development, which could help to improve the formation of a stronger dough structure and superior quality.

## Results

### Identification and characterization of seed proteins in CS and the *Glu-A3* deletion line CS-n

**Figure 1:**
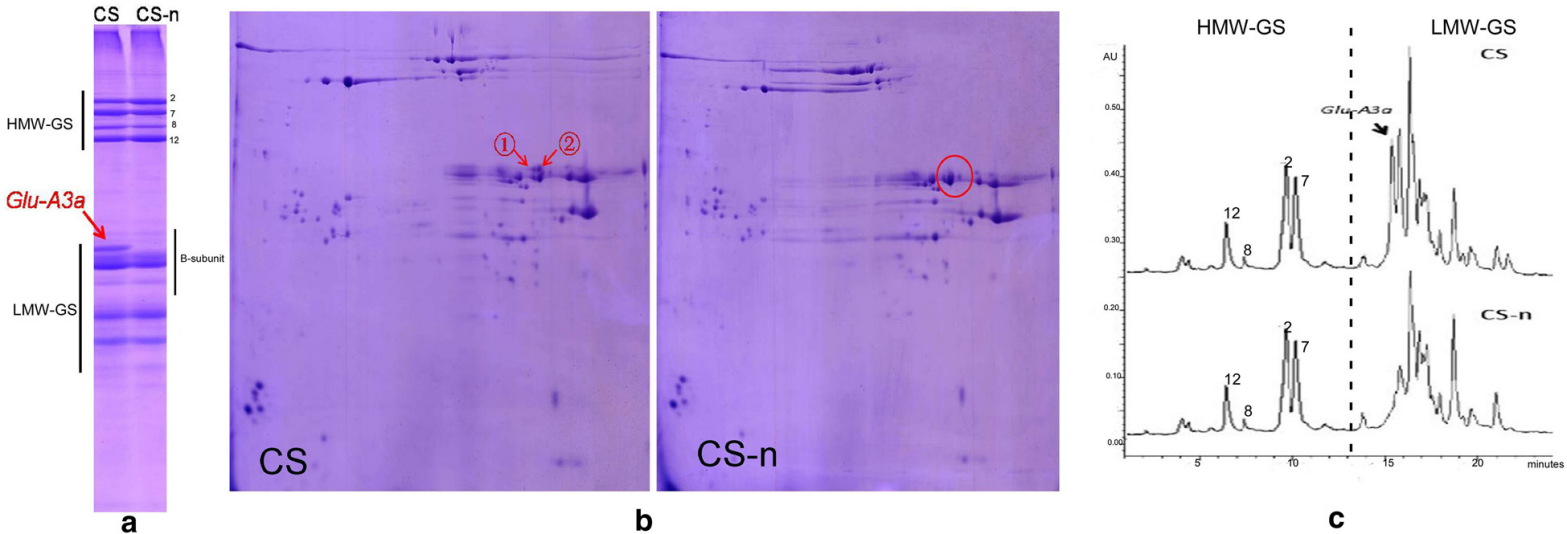
**Identification of** ***Glu-A3a*** **in Chinese Spring (CS) and** ***Glu-A3*** **deletion line CS-n. a**. SDS-PAGE: the *Glu-A3a* encoded B-subunit as well as LMW-GS and HMW-GS were indicated. **b**. 2-DE: two differentially expressed protein spots between CS and CS-n encoded by *Glu-A3a* were marked by ① and ②. **c**. RP-UPLC: two protein peaks encoded by *Glu-A3a* in CS as well as LMW-GS and HMW-GS were indicated*.*

A *Glu-3* deletion line of CS was screened and developed in our laboratory, and named CS-n. Compared to CS, the morphological characteristics of plants, spikes, and seeds, as well as the growth and development traits of CS-n showed no significant differences (Additional file 1: Figure S1, Additional file 2: Figure S2, and Additional file 3: Table S1). The grain protein compositions of CS and CS-n were identified by using various proteome approaches (Figure 1 and Additional file 4: Figure S3). The results indicated that CS-n showed the same albumin and globulin compositions as CS, while gliadins displayed minor differences between CS-n and CS; only one gliadin band obtained by acidic polyacrylamide gel electrophoresis (A-PAGE) was absent in CS-n (Additional file 4: Figure S3).

Glutenin subunits identified by SDS-PAGE indicated that HMW-GS in CS-n were the same as those in CS (N, 7 + 8, 2 + 12), and most LMW-GS bands were also identical, except that one clear B-type LMW-GS encoded by *Glu-A3a* was absent in CS-n (Figure 1a). Two-dimensional electrophoresis (2-DE) analysis revealed that *Glu-A3a* encodes two proteins (spots 1 and 2 in Figure 1b), which were further determined to be one LMW-i type subunit by liquid chromatography-tandem mass spectrometry (LC-MS/MS), as shown in Table 1. Reversed-phase ultra-performance liquid chromatography (RP-UPLC) analysis further confirmed that *Glu-A3a* encodes two protein components (peaks 1 and 2 in Figure 1c), which were eluted at 15.5 min and 16 min, respectively. Both peaks accounted for 22.58% of the total LMW-GS in CS.

**Table 1:** **LC-MS/MS analyses of peptides obtained after tryptic digestion of the isolated spot and bands**

**Protein origin**	**Identified sequences**	**Unique PepCount**	**Start**	**Stop**
Prokaryotic expression	VFLQQQCIPVAM	1735.0381	194	205
	VFLQQQCIPVAMQR	1719.0387	194	207
	SQMLQQSICHVMQQQCCQQLR	2693.0386	212	232
SDS-PAGE	VFLQQQCIPVAMQR	1735.0381	194	205
2-DE spots	MKTFLVFALLALAAA	1735.9338	1	15
	VFLQQQCIPVAMQR	1733.9270	214	227
	QIPEQSRHESIR	1479.7839	253	264
	QIPEQSR	857.4691	253	259
	TLPTMCSVNVPLYETTTSVPLGVGI	2649.4285	347	371

To obtain the accurate molecular mass of the *Glu-A3a*-encoded B-subunit, the expected protein band on the SDS-PAGE gel indicated in Figure 1a was collected and then analyzed by matrix-assisted laser desorption/ionization time-of-flight mass spectrometry (MALDI-TOF-MS). As shown in Additional file 5: Figure S4, the *Glu-A3a*-encoded LMW-GS B-subunit was easily identified, and its molecular mass was determined to be 41,701.2 Da.

### Confirmation of *Glu-A3a* deletion in CS-n with a sequence-tagged site polymerase chain reaction (STS-PCR) marker

**Figure 2:**
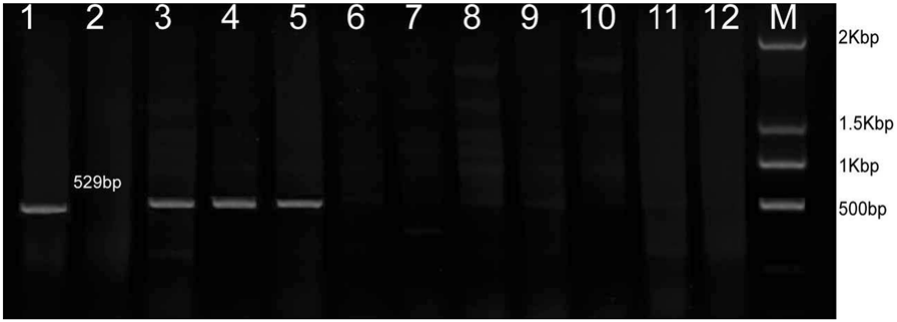
**Identification of** ***Glu-A3a*** **by STS-PCR markers. 1. CS (** ***Glu-A3a*** **), 2. CS-n; 3.** CS-1S^l^/1B; 4. CS 1S^l^ addition line; 5. Aroona-A3a (*Glu-A3a*); 6. Aroona-A3b (*Glu-A3b*); 7. Aroona (*Glu-A3c*); 8. Aroona-A3d (*Glu-A3d*); 9. Aroona-A3e (*Glu-A3e*); 10. Aroona-A3f (*Glu-A3f*); 11. Glenlea (*Glu-A3g*); 12. CB037A. M. molecular mass marker: 2000 bp, 1500 bp, 1000 bp and 500 bp. *Glu-A3a* fragment with 529 bp was arrowed.

To further confirm the deletion of the *Glu-A3* locus in CS-n, a pair of STS primers developed from the single nucleotide polymorphisms (SNPs) in *Glu-A3* allelic variants [24] were used to amplify the *Glu-A3a* gene. As shown in Figure 2, one specific PCR product of 529 bp was amplified in CS, the CS-1S^l^/1B substitution line, the CS-1S^l^ addition line, and Aroona, which contain the *Glu-A3a* allele, whereas no such fragments were obtained in the other materials without *Glu-A3a*, such as CS-n. The specific amplified 529-bp fragment was sequenced, and the sequence was the same as those from the upstream 140–395 bp of the *Glu-A3a*-coding sequence shown in Additional file 6: Figure S5. Thus, these results confirmed that the *Glu-A3* locus was deleted in CS-n.

### Comparison of gluten quality properties between CS-n and CS

Dough strength and breadmaking quality testing showed that the main gluten quality parameters in CS-n were significantly reduced compared to those of CS (Tables 2 and 3). In general, flour yield, water absorption, final viscosity, and peak viscosity between CS-n and CS showed no apparent differences. However, deletion of *Glu-A3a* in CS-n increased the ash content by 15.39%. Ash content is an important indicator of flour quality, which has a moderately negative effect on noodle color [25]. In addition, the deletion of *Glu-A3a* in CS-n resulted in a significant decrease of the gluten index (4% reduction) and an increase in the flour falling number (5.05% increase), as shown in Table 1. The gluten index was shown to have a positive relationship with strong dough property [26].

**Table 2:** **Quality parameters of dough and bread slices in CS and CS-n**

**Materials**	**Flour yield (%)**	**Ash (%)**	**Wet glutenin (%)**	**Total protein (%)**	**Water absorption**	**Development time (min)**	**Stability (min)**
CS	56.26	0.52 ± 0.01**	50.8 ± 0.5*	17.74	56 ± 0.1	4.7 ± 0.8**	11.4 ± 0.4*
CS-n	55.14	0.61 ± 0.01	52.1	17.68	56.4 ± 0.1	2.9 ± 0.4	9.3 ± 0.9
Materials	Tolerance index (FU)	Farinograph quality number	LV (cm^3^)	P/L of Alveograph NG Consistograph	Hardness (Force1)	Resilience sec (Area F-T)	Attenuation ratio (2:3)
CS	121 ± 5*	509 ± 5	770 ± 5**	0.25 ± 0.005**	628.4 ± 6.9*	7438.5 ± 326	64.02 ± 0.1
CS-n	95 ± 12	486 ± 11	735 ± 2.5	0.35 ± 0	586.87 ± 10.3	7093.6 ± 43	63.66 ± 0.6

**significant difference (P < 0.001), *means difference (P < 0.05).

**Table 3:** **Comparison of C-cell parameters of bread slices between CS and CS-n**

**Materials**	**Wrapper length**	**Slice brightness**	**Cell contrast**	**Number of cells**	**Cell density**	**Wall thickness**	**Cell diameter**	**Coarse/Fine clustering**	**Average cell elongation**	**Net cell elongation**
CS	1910 ± 4**	140.5 ± 1.8	0.747 ± 0.006	3163 ± 23	0.012178 ± 0.000138*	3.2 ± 0.03*	15.51 ± 0.66*	0.102 ± 0.018	1.78 ± 0.01^*^	1.33 ± 0.03^*^
CS-n	1857 ± 7	137 ± 0.4	0.747 ± 0.001	3086 ± 45	0.012287 ± 0.000128	3.07 ± 0.04	14.27 ± 0.06	0.077 ± 0.001	1.7 ± 0.02	1.23 ± 0.04

**Highly significant difference (P < 0.001), *Significant difference (P < 0.05).

**Figure 3:**
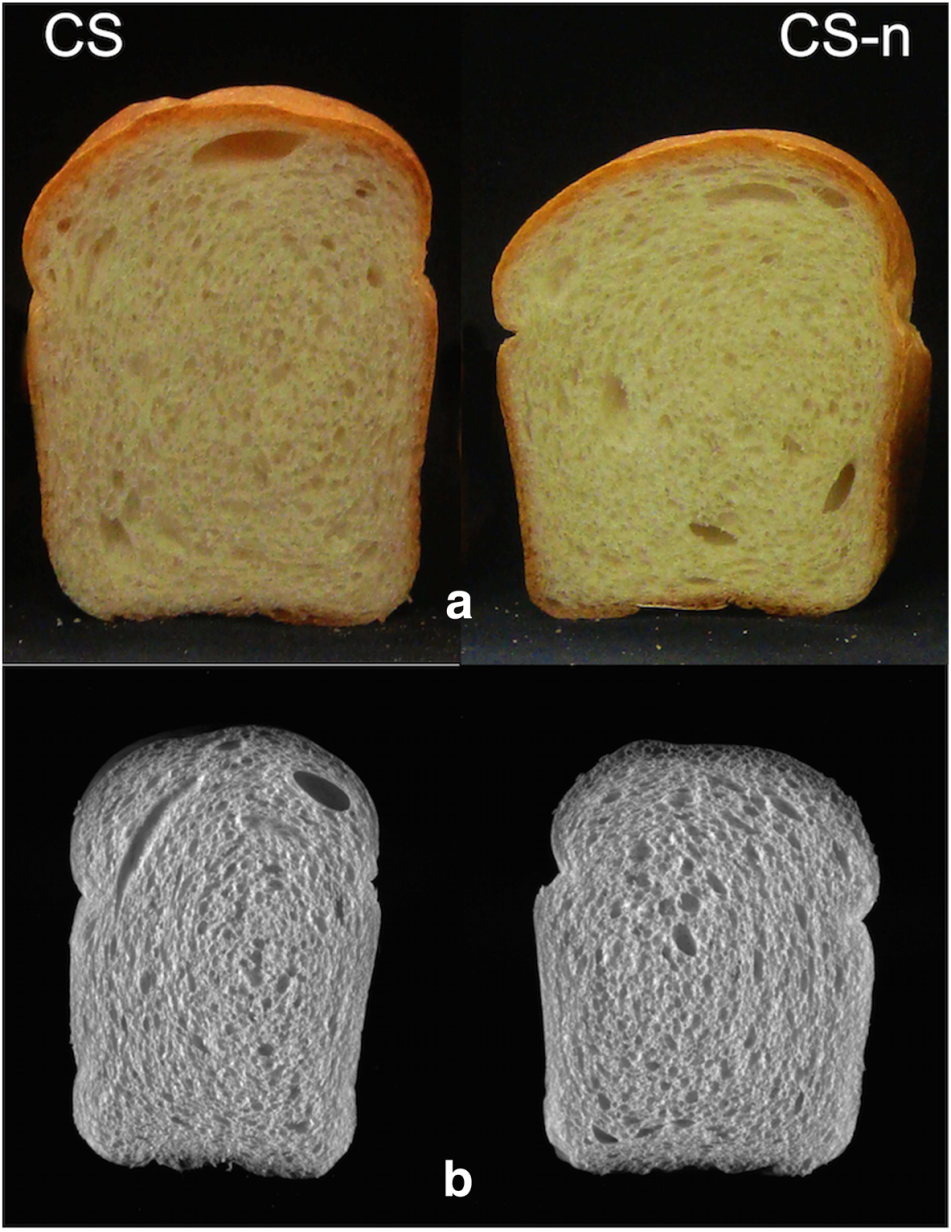
**The loaves baking pictures and C-cell pictures of CS, CS-n. (a)** The loaves baking pictures of CS and CS-n. **(b)** the C-cell pictures of CS and CS-n.

Farinograph analysis indicated that development time, stability time, tolerance index, and farinograph quality number in CS-n were significantly lower than those in CS (Table 2). These properties led to a decrease in loaf volume of CS-n from 760 to 735 cm^3^ (Table 2 and Figure 3). Bread texture analysis showed that the hardness and resilience of bread in CS were superior to those in CS-n. Further cell size analysis of the bread demonstrated that the quality in CS-n was significantly reduced (Table 3). For example, wrapper length, slice brightness, and wall thickness of CS-n bread slices were much lower than those of CS. The cell diameter and elongation in CS-n were also reduced as a result of *Glu-A3a* deletion.

### Molecular characteristics of the LMW-GS allele *Glu-A3a*

To further understand the molecular mechanisms underlying the significant effects of *Glu-A3a* on gluten and breadmaking quality, the complete coding sequence of *Glu-A3a* was amplified and sequenced by allelic-specific (AS) PCR. Based on the previously characterized *Glu-A3* genes, a pair of specific primers (A3-F and A3-R) for the *Glu-A3* locus was designed and used to amplify the *Glu-A3a* allele from CS. As shown in Additional file 7: Figure S6, a single band of approximately 1100 bp was obtained from CS, whereas no product was amplified from CS-n. Since most of the complete coding sequences of LMW-GS genes vary in length between 909 and 1167 bp [6,27-29], the size of the amplified band corresponded well to the known LMW-GS gene sizes. After sequencing of the amplified product, a complete open reading frame of 1134 bp was obtained. Sequence alignment showed that the cloned gene had no internal stop codons and contained typical structural features of LMW-GS, and therefore was named as *Glu-A3a* (Additional file 7: Figure S6). After searching the GenBank database, we found that the cloned *Glu-A3a* gene had the same sequence as *GluA3-11* from cultivar Aroona-A3a (GenBank accession number FJ549928). The deduced amino acid sequence of *Glu-A3a* showed the presence of an isoleucine as the first amino acid residue in the N-terminal of the mature protein, indicating that it belongs to the LMW-i type subunit [6].

The complete coding sequence of *Glu-A3a* was aligned with 15 other known LMW-i type genes to detect SNP and insertion/deletion (InDel) variations, and the results are listed in Table 4. These LMW-i genes originated from different *Triticum* species, including *T. aestivum* and *T. dicoccoides*. Six SNPs at different positions, resulting from G-A or C-T transitions and two deletions at nucleotides 81 and 854, were identified in *Glu-A3a*. Six SNPs could produce amino acid substitutions, and thus are considered nonsynonymous SNPs.

**Table 4:** **The positions of SNPs and InDels identified between** ***Glu-A3*** and other LMW-i type gene*

**LMW-GS**	**81-103**	**167**	**198**	**377**	**421**	**436**	**441**	**854**
FJ594428	-	T	G	T	T	C	A	-
Fifteen other LMW-i genes	CACCACCATTTTCGCAGCAACAACA	C	A	C	C/-	T/-	-	G

*Horizontal dashes indicated the deletions of nucleotide. Other 15 LMW-i genes included: 453157, AY453158, AY453159, AY453160, AY542896, AY831863, AY831865, AY831866, DQ217661, EU189087, FJ549931, FJ549932, FJ549933, FJ549934, JQ417918.

**Figure 4:**
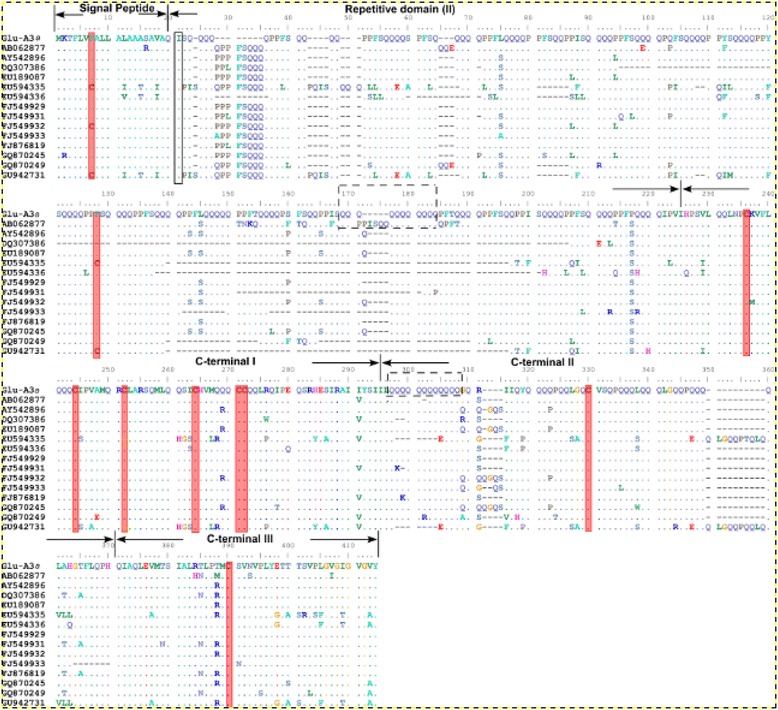
**Multiple alignment of the deduced amino acid sequences of** ***Glu-A3a*** **and other 14 LMW-i glutenin genes.** These genes including GenBank number AB062877 [14], AY542896 [13], DQ307386 [32], EU189087 [33], EU594335 and EU594336 [34], FJ549929, FJ549931, FJ549932 and FJ449933 [24], FJ876819 (Han, 2009), GQ870245, GQ870249 [35] and GU942731 [36]. Signal represents signal peptide (I), repetitive domain (II) and three sub-regions of C-terminal domain were indicated, respectively. The first amino acid residue of the mature proteins and cysteine residues were highlighted by black box and red shading, respectively. Deletions were indicated by dashes. Polyglutamine stretches were indicated by broken line frames.

The deduced amino acid sequence of *Glu-A3a* had 376 amino acid residues with a predicted molecular mass of 41,346.1 Da, corresponding well to that determined by MALDI-TOF-MS (41,701.2 Da). Multiple alignment of the deduced amino acid sequences of *Glu-A3a* with the other 14 LMW-i type subunits (Figure 4) showed that all have conserved signal peptides and four domains in the mature protein sequences, including a repetitive domain, cysteine-rich region, glutamine-rich region, and C-terminal conservative region, as reported by Cassidy et al. [27]. Similar to other LMW-i type subunits, the *Glu-A3a*-encoded subunit contained eight cysteine residues at relatively conserved positions (Additional file 8: Table S2). It is speculated that the first and seventh cysteines of the LMW-GS form the inter-molecular disulfide bond, while the rest form three intra-molecular disulfide bonds [30,31].

The number of repeats present in the repetitive domain is mainly responsible for the length variation and the general hydrophilic character of LMW-GS [30]. The *Glu-A3a*-encoded subunit contained the typical repeat motif of LMW-GS: P_1–2_FP/SQ_2–6_. Our results showed that *Glu-A3a* has a rather large and regular repeated sequence domain that includes a high proportion of glutamine residues (about 46%) in the repeats (consensus sequence PPFSQQQQ), and two polyglutamine stretches with 11 and 12 continuous glutamine residues in the repetitive and C-terminal domains, respectively. Repeat motif numbers in LMW-i subunits are much higher than those in the LMW-m and LMW-s subunits, ranking them the longest protein subunits among all *Glu-3* loci.

### Secondary structure and function prediction of the *Glu-A3a*-encoded protein

The secondary structures of the *Glu-A3a*-encoded protein (FJ549928) and five other LMW-i type subunits from bread wheat (AY724436, AY724437, AY263369, AY831866, and AY542896) were predicted by the PSIPRED server, as shown in Table 5. The results showed that the α-helices and β-strands were dispersed in the normal configuration in C-terminal I and were highly conserved in C-terminal III. FJ549928 contained seven α-helices, mainly located at the C-terminal, and one β-strand dispersed in the conserved C-terminal region. Thus, the number of α-helices in FJ549928 was much higher than that of the other five subunits, which contain only 0–3 α-helices. For example, the LMW-i type glutenin subunit AY542896, assigned to the 1A chromosome, only has one α-helix, which was confirmed to co-migrate with the LMW-50 subunit that plays an important role in determining good quality characteristics of *Glenlea* [13] and the XYGluD3-LMWGS1 subunit (AY263369), with only 3 α-helices, is also considered to have a positive effect on dough quality [37].

**Table 5:** **The secondary structure prediction of the six deduced LMW-GS**

**LMW-GS**	**Structure motifs**	**Contents (%)**	**Total**	**Dispersal in every region**				
				**N-terminal domain**	**Repetitive domain**	**C-ter domain I**	**C-ter domain II**	**C-ter domain III**
AY724436	α-helix	-	-	-	-	-	-	-
	β-strand	1.32	2	-	-	-	-	2
Y724437	α-helix	10.51	3	-	-	2	-	1
	β-strand	0.68	1	-	-	-	-	1
AY263369	α-helix	11.6	3	-	-	2	-	1
	β-strand	1.4	2	-	-	1	-	1
AY831866	α-helix	5.98	3	-	-	2	-	1
	β-strand	1.09	2	-	-	-	1	1
AY542896	α-helix	2.72	1	-	-	-	-	1
	β-strand	1.09	2	-	-	1	-	1
*Glu-A3a*	α-helix	15.87	7	-	1	5	1	-
	β-strand	0.79	1	-	-	-	-	1

### Phylogenetic analysis of *Glu-A3a* and other LMW-GS genes

**Figure 5:**
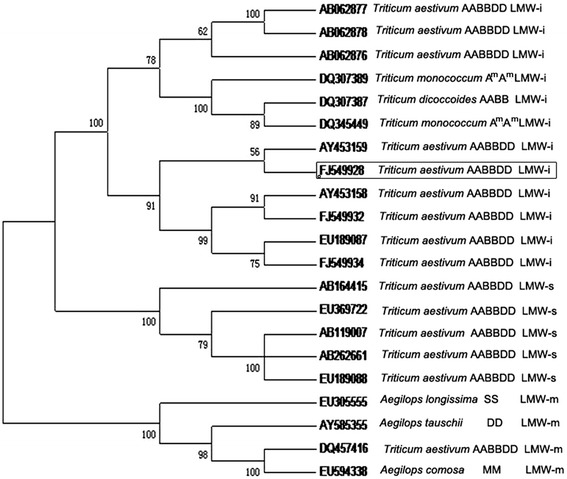
**Homology tree constructed based on the coding regions of 21 LMW-GS genes.** 21 LMW-GS genes named AB062876, AB062877 and AB062878 [14], AB262661 (Takeuchi T, 2006), AB119007 and AB164415 [40], AY453158 and AY453159 [41], AY585355 [42], DQ307389, DQ307387 and DQ345449 [39], DQ457416 [43], EU305555 [44], EU594338 [34], EU189087 and EU189088 [33], FJ549928, FJ549932 and FJ549934 [24]. The suffixes of GenBank accession numbers indicated the different types of the genes. *Glu-A3a* gene was circled by frame.

A homology tree was constructed to reveal the phylogenetic relationships among 25 LMW-GS genes at *Glu-3* loci from different species and genomes through nucleotide sequence alignment of their coding regions using MEGA5 software (Figure 5). These sequences comprised 21 LMW-GS genes from different genomes of *Triticum* diploid, tetraploid, and hexaploid species. The phylogenetic tree displayed two clear branches, which corresponded well to distinguishing the LMW-i type from the LMW-m and LMW-s type subunits. This demonstrated that LMW-i type genes have undergone greater divergence during evolution compared to LMW-s and LMW-m genes, as previously reported [38,39]. Sine LMW-m and LMW-s type subunit genes generally show higher consistency, they showed close phylogenetic evolutionary relationships. *Glu-A3a* showed a closer relationship with other LMW-i type genes from common wheat. All of the LMW-i type subunit genes from common wheat and related species shared higher sequence identity, indicating their high evolutionary conservation.

### Heterologous expression of *Glu-A3a* in *Escherichia coli* and determination of the corresponding native protein encoded by *Glu-A3a*

**Figure 6:**
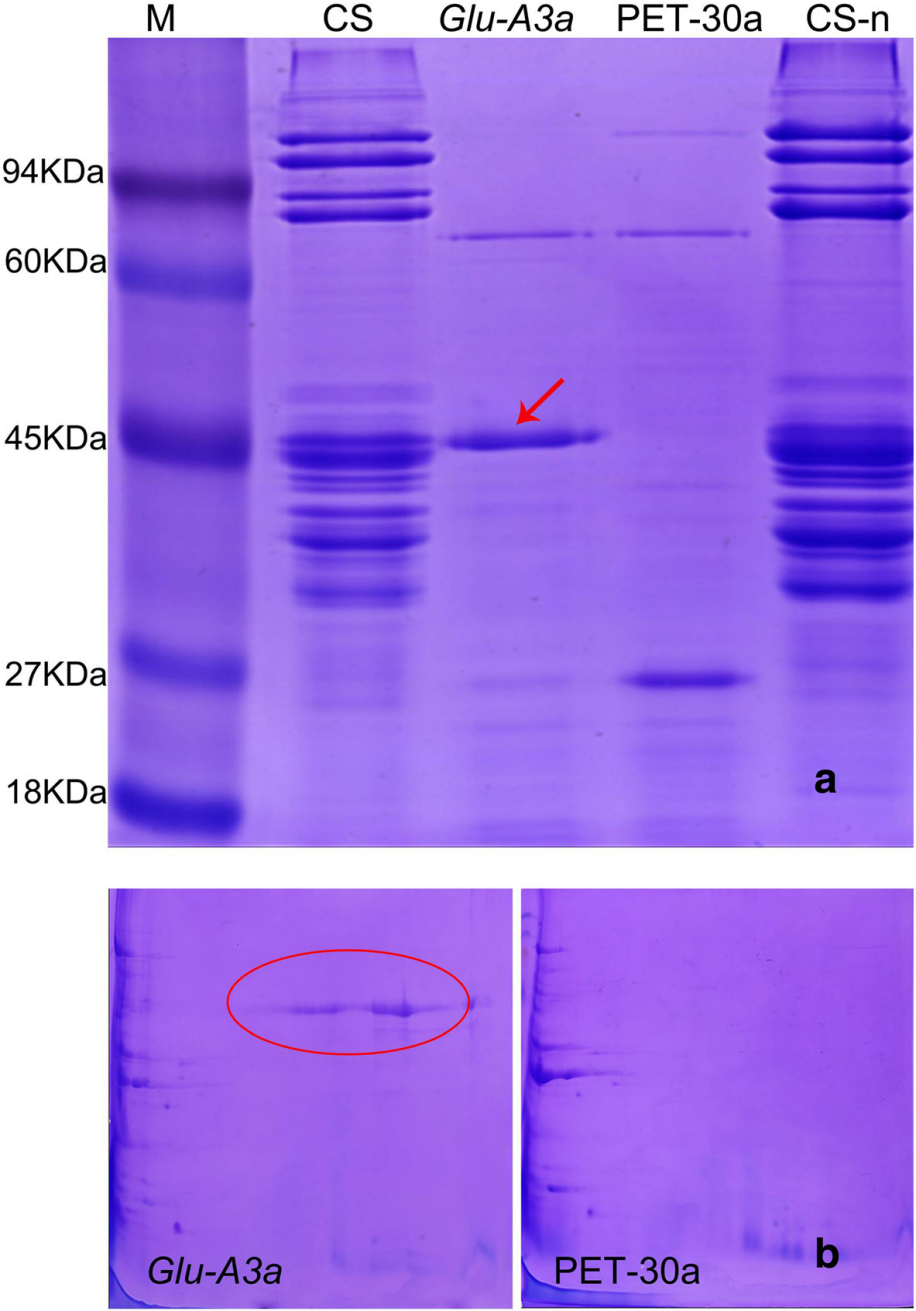
**Identification of heterologous expressed protein of** ***Glu-A3a*** **in** ***E. coli*** **by SDS-PAGE (a) and 2-DE (b). (a)** The SDS-PAGE of the heterologous express protein of *Glu-A3a.* M is the protein marker (94 kD, 60 kD, 45 kD, 27 kD, 18 kD), CS is the gluten of CS, *Glu-A3*a is the heterologous express protein, PET-30a is the vector, CS-n is the gluten of it. The *Glu-A3a* expressed protein was indicated by red arrow. **(b)** The 2-DE picture of the heterologous express protein and the vector PET-30a, the difference was marked by red circle.

The *Glu-A3a*-coding region without signal peptides was expressed in *E. coli*. The expressed fusion protein was separated by both SDS-PAGE and 2-DE, and was further identified by LC-MS/MS. SDS-PAGE identification (Figure 6a) indicated that the relative mobility of the expressed protein was the same as that of the native *Glu-A3a-*encoded subunit of CS, confirming that *Glu-A3a* without the N-terminus can be expressed normally, similar to other LMW-i type genes [13]. Furthermore, 2-DE separation of the expressed protein (Figure 6b) demonstrated a similar pattern as that shown in Figure 1b. LC-MS/MS identification also confirmed that the expressed protein was the *Glu-A3a*-encoded subunit present in CS, as revealed by the previous tandem MS results (Table 1).

To verify the authenticity of the cloned sequence, LC-MS/MS was conducted by using the native *Glu-A3a* subunit digested by trypsin. We compared the results of LC-MS/MS of the SDS-PAGE band of CS, the heterologous protein, 2-DE spots, and the amino acid sequence of the *Glu-A3a* gene. This gives a coverage rate of 18.26% (65/356 amino acids of the mature polypeptide). These results revealed consistency in the peptide sequences among the samples, confirming the correspondence of the *Glu-A3a* gene and its native encoded subunit.

### Dynamic expression profiles of the *Glu-A3a* gene and its encoded protein during grain development

**Figure 7:**
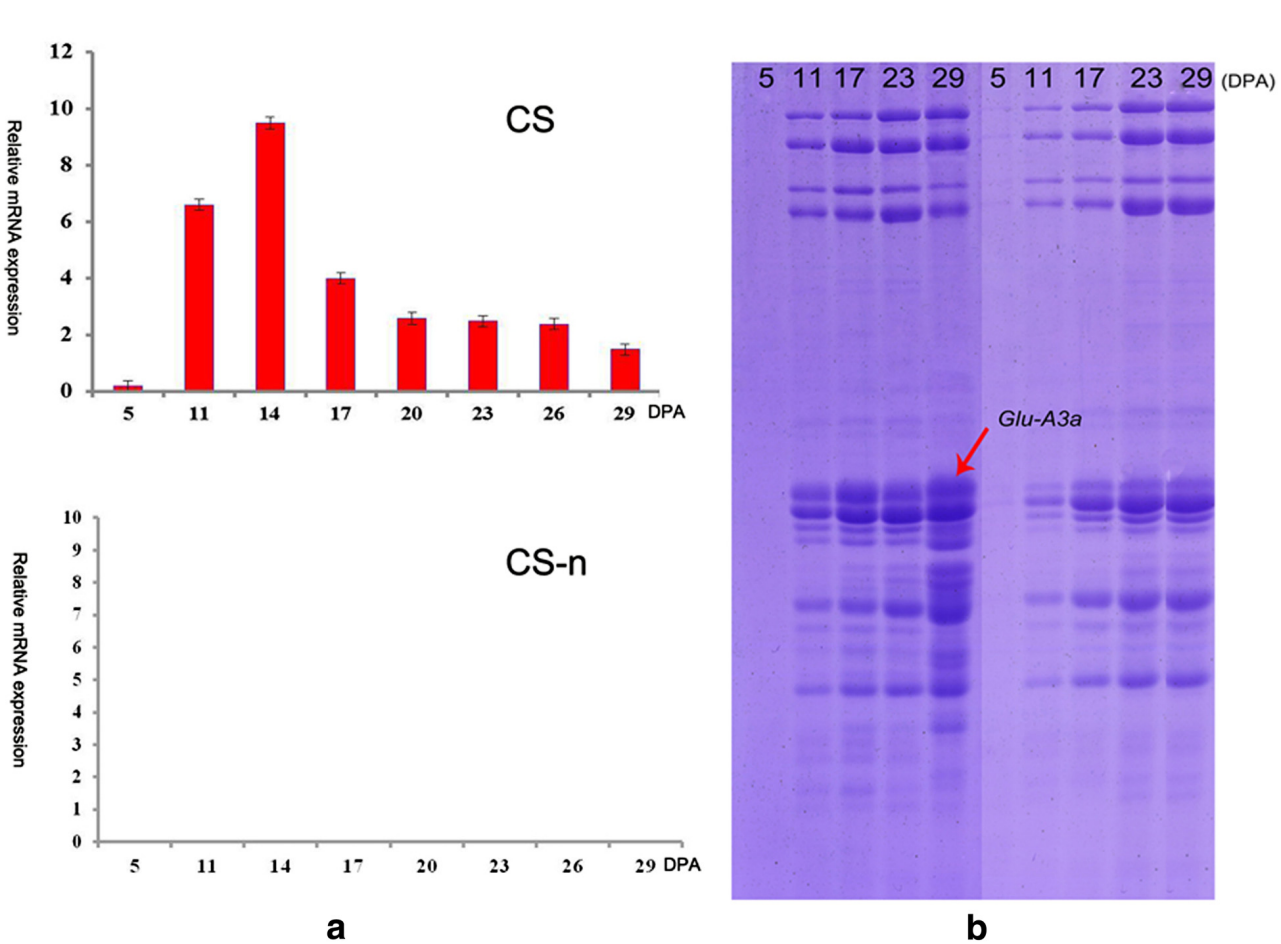
**Expression patterns of** ***Glu-A3a*** **gene and its encoding protein. (a)** Expression patterns of *Glu-A3a* gene during grain development (5, 11, 14, 17, 20, 23, 26, 29 DPA) of CS and CS-n by qRT-PCR. **(b)** The SDS-PAGE of the subunit *Glu-A3a* of 5, 11, 17, 23, 29 DPA. The *Glu-A3a* encoded subunit in CS was arrowed.

The dynamic transcription expression profiles of the *Glu-A3a* gene at 5, 11, 14, 17, 20, 23, 26, and 29 days post anthesis (DPA) of grain development were detected by quantitative real-time (qRT)-PCR in both CS and CS-n. Real-time melting temperature curves for the gene showed a single peak. qRT-PCR efficiency was determined by five serial five-fold dilutions of cDNA, and the standard curve confirmed high RT-PCR efficiency rates (Additional file 9: Figure S7). As shown in Figure 7a, the *Glu-A3a* gene displayed an up-down expression pattern during grain development of CS, with peak expression occurring at 14 DPA. However, *Glu-A3a* mRNA could not be detected in CS-n, further confirming the deletion of the *Glu-A3* locus. SDS-PAGE analysis showed that the *Glu-A3a*-encoded B-subunit exhibited a gradual up-regulated expression pattern, and it began to rapidly accumulate after 11 DPA (Figure 7b). At 5 DPA, no LWM-GS genes could be detected, and both LMW-GS and HMW-GS showed trace expression levels. After 14 DPA, the B-subunit as well as other LMW-GS and HMW-GS genes displayed significant up-regulation, and peak expression occurred at 17 DPA (Figure 7b).

### Development and validation of an SNP-based molecular marker for *Glu-A3a*

**Figure 8:**
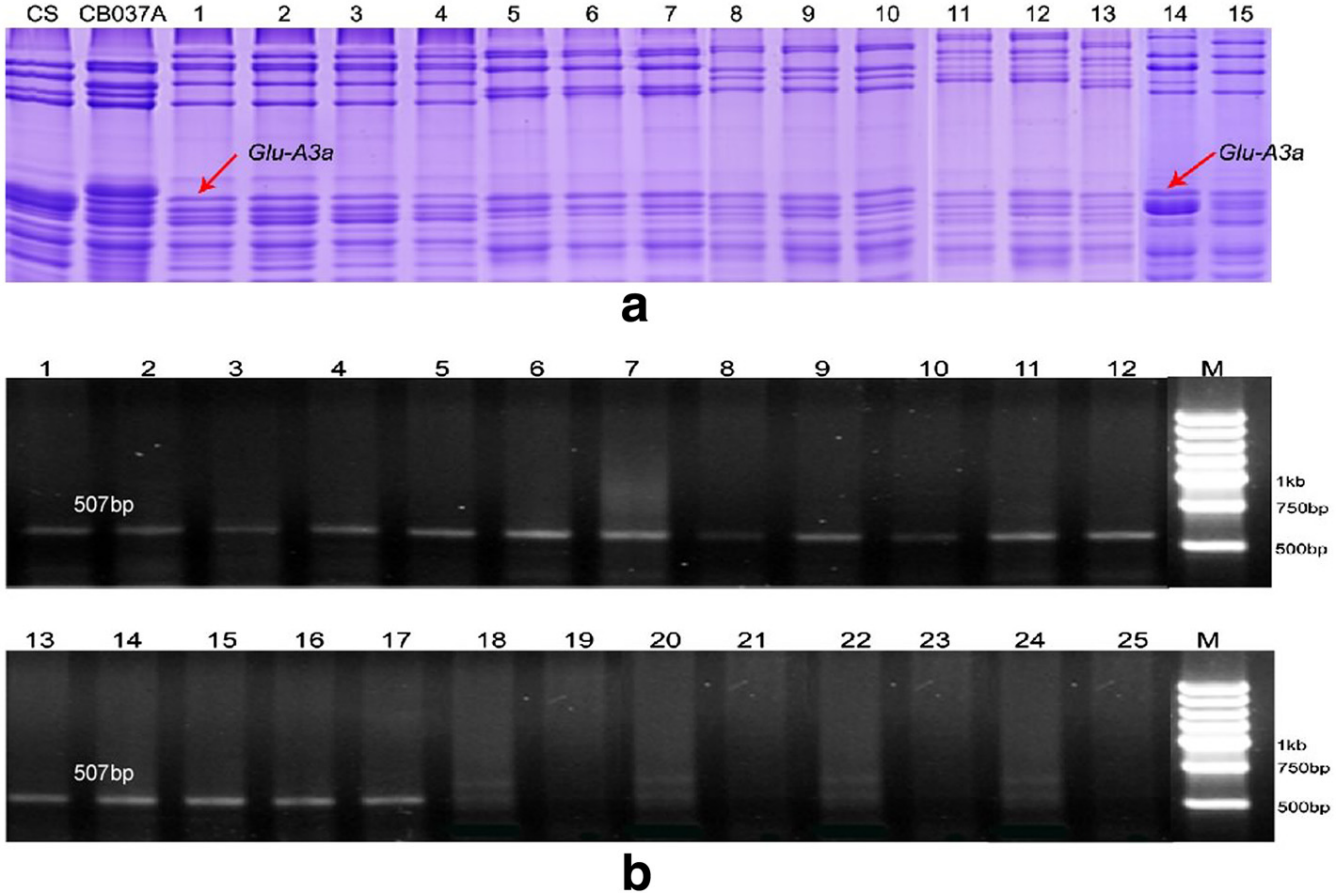
**Development and validation of SNP-based molecular marker for** ***Glu-A3a*** **. (a)**. SDS-PAGE of glutenin subunits: RIL (1–13), CS, CB037A, CS-1S^l^/1B (14) and Chinese Spring S genome addition line (15). **b**. PCR amplification from bread wheat cultivars with different *Glu-A3* allele compositions, RIL (1–13), CS-1S^l^/1B (14), Chinese Spring S genome addition line (15), CS (16) and Aroona-A3a (17). 18–25 are Aroona-A3b (*Glu-A3b*), Aroona (*Glu-A3c*), Aroona-A3d (*Glu-A3d*), Aroona-A3e (*Glu-A3e*), Aroona-A3f (*Glu-A3f*), Glenlea (*Glu-A3g*), CB037A and CS-n, respectively. M is marker (5 kb, 4 kb, 3 kb, 2 kb, 1 kb, 750 bp and 500 bp).

An AS-PCR marker was developed based on the SNPs detected in *Glu-A3* genes. A pair of specific primers for *Glu-A3a* (*Glu-A3a* F: GCAAAGAAGGAAAAGA GGTGG, R: GGTTGTTGTTGTTGCTGCA) was designed and tested in different genotypes and hybrid generations with different *Glu-A3* alleles. The materials with different *Glu-A3* alleles included 48 bread wheat cultivars, the CS-1S^l^/1B substitution line, and the CS-1S^l^ addition line, as well as seven Aroona NILs and four recombinant inbred lines (RILs) derived from a cross between the CS substitution line CS-1S^l^/1B with *Glu-A3a* and the bread wheat cultivar CB037A with *Glu-A3c* (Additional file 10: Table S3). The *Glu-A3* allele compositions of all materials used were identified by SDS-PAGE (Figure 8a). The PCR results showed that one specific PCR product of 507 bp was amplified in all cultivars with *Glu-A3a* (Figure 8b). To validate the effectiveness of the STS marker, seven NILs and four RILs with different *Glu-A3* allele compositions were used for PCR amplification. The results showed that the 507-bp fragment could be specifically amplified in the lines with the *Glu-A3a* allele, whereas no any amplification products were obtained from the lines with other *Glu-A3* alleles, including CS-n without the *Glu-A3* locus. These results confirmed that the developed AS-PCR marker could be used as an effective tool for rapidly screening the *Glu-A3a* allele in wheat quality improvement strategies through molecular marker-assisted selection.

## Discussion

In the present study, we performed a comprehensive survey on the molecular characteristics of *Glu-A3a* from a *Glu-A3a*-deletion line (CS-n), using proteomic and molecular biological methods. Here, we focus our discussion on the allelic variations at *Glu-A3* loci, the structure and expression features of *Glu-A3a*, and molecular marker discovery and its potential application in wheat quality improvement.

### Allelic variations at *Glu-3* loci and their effects on gluten quality

LMW-GS account for approximately 60% of glutenin proteins in mature seeds and play important roles in the formation of glutenin macropolymer and gluten quality [1,45], particularly for dough extensibility and strength [3-6,17]. LMW-GS genes belong to a multiple gene family and are found in multiple copies in *Triticum aestivum*; the copy number in hexaploid bread wheat was estimated to vary from 10–15 [46] to 35–40 [27,47]. A recent study based on BAC library screening and proteomics analysis showed that *Glu-A3*, *Glu-B3*, and *Glu-D3* in the Chinese bread wheat cultivar Xiaoyan 54 contain 4, 3, and 7 genes, respectively [16]. In addition, by using the LMW-GS gene marker system, at least 15 LMW-GS genes were identified in Aroona NILs [17].

*Glu-A3* and *Glu-B3* alleles are known to play a major role in determining differences in processing qualities among the three *Glu-3* loci, while *Glu-D3* alleles play minor roles in determining quality variation in bread wheat [17]. In particular, the *Glu-A3* locus was considered to have the biggest contribution to quality among all LMW-GS loci, in which *Glu-A3f* was found to have a strong positive effect on end-use quality [48]. In Australian wheat cultivars, LMW-GS provided better predictions of Rmax than HMW-GS [45]. The effects of different *Glu-3* alleles on Rmax showed the following ranking: *Glu-A3b* > *Glu-A3d* > *Glu-A3e* > *Glu-A3c*, *Glu-B3i* > *Glu-B3b* = *Glu-B3a* > *Glu-B3e* = *Glu-B3f* = *Glu-B3g* = *Glu-B3h* > *Glu-B3c*, and *Glu-D3e* > *Glu-D3b* > *Glu-D3a* > *Glu-D3c* > *Glu-D3d* [17]*.* However, no studies of the effect of the *Glu-A3a* allele on gluten quality have been reported so far. In the present work, we found that the deletion of *Glu-A3a* significantly reduced dough strength and breadmaking quality, including most of the mixing and bread quality parameters (Tables 2 and 3). This indicates that *Glu-A3a* plays important roles in conferring high gluten quality to wheat.

### Molecular basis of the relationship between *Glu-A3a* and gluten quality

The molecular structures of LMW-GS proteins play important roles in determining the dough strength and gluten quality; in particular, the distribution of cysteine residues could lead to functional protein differences [6]. The first and the seventh cysteines form the inter-molecular disulfide bond, while the remaining cysteines form three intra-molecular disulfide bonds [11,30,31]. Thus, the number and position of cysteines are important to the formation of the secondary protein structure and, consequently, dough quality. The presence of a long repetitive domain is also considered to have a positive influence on wheat flour quality [30,49]. A repeated sequence domain could be helpful in increasing the viscosity and elasticity of the dough through increasing the inter-molecular interactions among the large number of glutamine side chains, which are both good hydrogen bond donors and acceptors [49-51]. According to Masci et al. [30], all the predicted α-helices in the 42 K LMW-GS seem to be located near the intra-molecular disulfide bonds. They also speculated that helix-helix interactions are involved in guiding the formation of the intra-molecular disulfide bonds. Therefore, a higher α-helix content may contribute to better quality of the dough [52]. The β-strands are generally considered to endow the protein with high elasticity and to improve the capability to resist distortion [38].

LMW-i type subunits contain eight highly conserved cysteine residues in the C-terminal domain (Table 5), which differ from the LMW-m and LMW-s subunits that have a cysteine residue in the N-terminus or in the repetitive domain [39]. Therefore, the secondary structures of LMW-i subunits are expected to be quite different from those of the LMW-m and LMW-s subunits. Previous work on LMW-GS AY542896 and AY263369 indicated that they have positive effects on quality properties [13,37]. In the present study, comparative analysis with the secondary structures of AY542896 and AY263369 showed that the *Glu-A3a*-encoded subunit had more α-helices (Table 4). The secondary structure is the foundation for a highly complex spatial conformation and is composed of structural motifs, including α-helices, β-strands, and random coils. The higher α-helix number in the *Glu-A3a*-encoded subunit could guide the formation of the intra-molecular disulfide bonds and contribute to superior dough strength and breadmaking quality.

The sizes of most of the cloned LMW-GS genes range from 900 to 1000 bp, and the gene *LMW-TD22* with 1167 bp is the longest complete gene among the cloned LMW-i genes analyzed to date [29]. The presence of a long repetitive domain is considered to have a positive influence on wheat flour quality because it can form more β-turns in the structure, thereby conferring elasticity to the protein molecule [30,52]. The molecular structure of the deduced LMW-TD22 subunit indicated a long repetitive domain of 21 repeat motifs (consensus sequence P_1–2_FP/SQ_2–6_). In the present study, the *Glu-A3a*-encoded subunit was also found to have a long repeated sequence domain and a high proportion of glutamine residues (about 46%), which could improve the conformation of superior gluten structure and breadmaking quality.

### *Glu-A3a* expression and LMW-GS synthesis

It is well known that the expression levels as well as accumulation patterns of storage proteins are closely associated with gluten quality properties [53-55]. For example, the over-expressed HMW-GS 1Bx7^OE^ has positive effects on dough strength [53,56]. In addition, the accumulation rates vary between different groups of proteins, suggesting differential regulation of protein biosynthesis and different quality performance. In particular, the wheat biotype with superior HMW-GS 5 + 10 subunits accumulated larger polymers more quickly than the biotype with poor allelic subunits 2 + 12 [54].

The B-subunits of LMW-GS are the most abundant and have the greatest impact on wheat processing qualities [6]. In this work, RP-UPLC analysis revealed a higher expression level and greater proportion of *Glu-A3a*-encoded B-subunits, accounting for more than 22% of the total LMW-GS in CS (Figure 1c), indicating its major contribution to LMW-GS synthesis and its important roles in determining dough quality. A recent study also found that higher numbers of active LMW-GS genes at the *Glu-A3* and *Glu-D3* loci in Xiaoyan 54 tended to produce greater ZSVs, an important indicator of breadmaking quality [16]. Similarly, the decrease in the number of active LMW-GS genes in CS-n due to deletion of the *Glu-A3* locus likely contributed to the significant reduction in dough strength and breadmaking quality (Table 2 and 3).

Wheat glutenin proteins generally display an up-regulated expression pattern during grain development (Figure 7a). Coordinated accumulation of transcripts from HMW-GS and LMW-GS genes, as well as α-, γ-, and ω-gliadin genes, occurs early in grain development [54,57]. LMW-GS, HMW-GS, and ω-gliadins can be detected by gel electrophoresis as early as 7 DPA [54], and 10–18 DPA represents the key stage of storage protein synthesis [58]. In the present study, *Glu-A3a* transcripts demonstrated an up-down expression pattern during grain development, and the highest expression level occurred at 14 DPA (Figure 7a), similar to a previous report [59]. SDS-PAGE analysis revealed that the *Glu-A3a*-encoded B-subunit displayed an up-regulated expression pattern and showed rapid synthesis and accumulation at 11–17 DPA (Figure 7b), which is also generally in agreement with a previous report [55]. Thus, the *Glu-A3a*-encoded B-subunit has a higher accumulation rate during grain development similar to HMW-GS 5 + 10 [54], which could improve the conformation of the regular gluten structure. Some important genes related to storage protein folding and synthesis, such as protein disulfide isomerase (PDI) and binding protein (BiP) genes, generally have higher expression levels at the early grain developmental stages. For instance, the PDI genes *PDIL1-1* and *PDIL2-1*, which are involved in disulfide bond formation, displayed a peak expression level in the early stages (about 10–15 DPA) of grain development [58]. The higher accumulation rate of the *Glu-A3a*-encoded B-subunit was accompanied by higher expression levels of the genes involved in storage protein synthesis and assembly during early grain development, suggesting that this subunit could improve the conformation of gluten macropolymers (GMP) and result in superior dough quality.

### Potential application of *Glu-A3a* in wheat quality improvement through molecular marker-assisted selection

Characterization of the allelic variations of LMW-GS is important for improvement of wheat-processing quality. Some allelic variations of LMW-GS have greater positive effects on dough properties than others [3,45,60]. Marker-assisted selection is an effective supplement to conventional breeding practices. For LMW-GS, because of the low resolution of traditional SDS-PAGE and the tedious operation procedures involved in 2-DE, development of different markers is important for the study and application of target subunits.

Recently, with increasing numbers of LMW-GS alleles being cloned and sequenced from common wheat, different molecular markers have been developed to rapidly screen and select desirable *Glu-3* alleles. Zhang et al. [41] developed a set of markers that can be used to discriminate the alleles *Glu-A3a, b, c, d, e, f,* and *g.* Long et al. [61] classified 69 LMW-GS genes registered in GenBank into nine groups and established nine group-specific primer sets to identify each group. Ikeda et al. [62] developed 12 specific PCR markers to distinguish 12 groups of LMW-GS genes in the wheat cultivar Norin 61. Ten allele-specific STS markers for *Glu-D3* were developed by Zhao et al. [43,63,64]. Wang et al. [35] designed 10 allele-specific PCR markers for the *Glu-B3* locus based on SNPs present in the sequences of different allelic variants. Wang et al. [24] reported an allele-specific marker for *Glu-A3b*, which was not reported by Zhang et al. [41].

In the present study, we developed a new allele-specific PCR marker that can effectively discriminate *Glu-A3a* from other *Glu-A3* alleles, which was validated using different cultivars, including RILs and NILs (Figure 8b). This *Glu-A3a* allele-specific marker can be used in marker-assisted breeding strategies aimed at the improvement of wheat quality. With help of this marker, it will be very convenient and effective for breeders to select this superior gene in early hybrid generations of a wheat quality program. However, this marker can only identify one specific gene in different generations and materials one time. The multiplex PCR systems showed to be more rapid and economic in identifying different desirable genes [24]. Thus, to improve the selection efficiency, it is needed to further develop multiplex PCR markers that can rapidly identify different desirable genes, including *Glu-A3a* and other quality-related genes.

## Conclusions

In the present study, we carried out the first molecular characterization and functional analysis of the properties of the *Glu-A3a* allele by using a *Glu-A3* deletion line of the CS wheat variety (CS-n). The deletion of *Glu-A3a* had no clear effects on plant morphological and yield traits, but significantly reduced gluten strength and breadmaking quality. Molecular characterization revealed that *Glu-A3a* contains 1134 bp encoding one LMW-i type B-subunit that had longer repetitive domains, an increased number of α-helices, and showed a higher expression level and accumulation rate during grain development. These features could explain its major role in the formation of dough strength and breadmaking quality and indicate its potential value for wheat quality improvement. A specific AS-PCR marker for the *Glu-A3a* allele was developed and validated using different bread wheat cultivars, NILs, and RILs, which could be used as an effective molecular marker for gluten quality improvement through marker-assisted selection.

## Method

### Plant materials

Chinese Spring (*Triticum aestivum* L., 2n = 6x = 42, AABBDD) and its *Glu-A3a* deletion line (CS-n) developed in our laboratory were used in this study. Aroona-A3a (*Glu-A3a*) and its six NILs: Aroona-A3b (*Glu-A3b*), Aroona (*Glu-A3c*), Aroona-A3d (*Glu-A3d*), Aroona-A3e (*Glu-A3e*), Aroona-A3f (*Glu-A3f*), and Glenlea (*Glu-A3g*) were used for identifying *Glu-A3* alleles, which were kindly provided by Dr. Xianchun Xia, Institute for Crop Science, Chinese Academy of Agricultural Science (CAAS). Bread wheat cultivars CB037A, Chinese Spring substitution line CS-1S^l^/1B [58], Chinese Spring 1S^l^ genome addition line, and four recombination inbred lines (RILs) produced by crossing between CB037A and Chinese Spring substitution line CS-1S^l^/1B were used for identifying *Glu-A3a* deletion in CS-n, and developing and validating AS-PCR marker for *Glu-A3a*. All materials used in this study were listed in Additional file 10: Table S3.

### Identification of seed proteins

#### Protein extraction, A-PAGE, SDS-PAGE and RP-UPLC

According to the solubility in a series of solvents, grain albumins, globulins, gliadins and glutenins were extracted according to the established methods [65,66].

#### A-PAGE

A-PAGE was conducted based on the method of Yan et al. [66,67].

#### SDS-PAGE

SDA-PAGE was performed with Bio-Rad PROTEAN II XL equipment based on the previously described method [68] with 12% gel and electrophoresed at 15 mA for 2 h.

#### RP-UPLC

RP-UPLC was used to separate HNW-GS and LMW-GS based on the recent reports [56,69]. The samples were performed on an Agilent 1100 using a Zorbax 300SB-C18 column (300 A° pore size and 5 mm particle size).

#### MALDI-TOF-MS

MALDI-TOF-MS was used to detect the accurate molecular weight (MW) of LMW-GS according to the previous reported method [39,70]. Shimadzu corporation AXIMA-CFRTM Plus MS apparatus (Japan) and the matrix of sinapinic acid (SA, a-cyano-4-hydroxycinnamic acid) were used.

#### 2-DE (IEF X SDS-PAGE)

Grain glutenins and heterologously expressed LMW-GS were separated and identified by 2-DE (IEF × SDS-PAGE). The first dimension was performed by an EttanTM IPG-phor II TM system (GE Healthcare, USA) using 18 cm strips (pH 6–11). The IEF rehydration solution was 7 M urea, 2 M thiourea and 4% CHAPS. The rehydrate condition was 30 V at 20°C for 12 h while the IEF condition was 300 V for 1 h, 500 V for 1 h, 1000 V for 1 h, 3000 V for 1 h, and 8000 V to 80,000 V for 10 h. The second dimension was performed on a 12% acrylamide gradient. After electrophoresis, the 2-DE gels were stained with colloidal Coomassie Brilliant blue (CBB) (R-250/G-250 = 4:1) and analyzed by using ImageMaster™ 2-D platinum software version 5.0 (Amersham Bioscience, Swiss Institute of Bioinformatics, Geneva, Switzerland, 2003) based on Lv et al. [71] with minor modifications. Three biological replicates were performed.

#### LC-MS/MS

The grain native and heterologous expressed LMW-GS separated by SDS-PAGE and 2-DE were further identified by LC-MS/MS. The expected LMW-GS band on the SDS-PAGE gel and 2-DE spots were excised and digested with trypsin according to Jin et al. [72]. The digested protein (0.5 ml) was subject to MS analysis in a Waters SYNAPT High Definition Mass Spectromet ry™ (HDMS) mass spectrometer. The software BioworksBrowser 3.3 was used to analyze the LC-MS/MS data.

### Gluten quality testing

Both CS and its CS-n were planted in Beijing experimental station of Chinese Academy of Agricultural Sciences during 2013–2014 growing season. The field design was three replications and each blot was 20 m^2^. Gluten quality parameters were tested according to Sun et al. [73] at Academy of State Administration of Grain. Flour moisture and ash contents (% dry basis) were determined according to the American Association of Cereal Chemists Approved Methods (2000) 44-15A and 08–02, respectively. Protein content (%N 5.7, 14% moisture basis) was determined by nitrogen combustion analysis with a LECO (Model FP analyzer, St. Jopeph, MI) calibrated against EDTA.

Farinograph parameters were obtained by using 10 g Brabender Farinograh-E based on American Association of Cereal Chemists Approved Method (2000) 54–21.

Image analysis of crumb grain of bread was performed with a C-Cell, image analysing software and equipment (Calibre Control International Ltd.). Slice brightness and cell contrast were used to describe the brightness of slices. Number of cells, wall thickness, cell diameter, cell volume, coarse cell volume and average cell elongation were used to measure the cell properties. The sliced samples for textural analysis were prepared in the same way as those for the C-Cell.

### DNA extraction and PCR amplification

Total genomic DNA from dry seeds was extracted according to McDonald et al. [74] and An et al. [39] with minor modifications. A pair of AS-PCR primers (A3-F and A3-R) was designed to amplify the coding regions of LMW glutenin gene based on the previously cloned sequences [13,24]. The primer sequences were A3-F: 5’-GCCTTTCTTGTTTACGGCTG-3’, A3-R: 5’-TCAGATTG ACATCCACACAAT-3’ (synthesized by Sangong Inc., China). PCR amplifications were performed in 50 μl reaction volumes containing 2.5 U La Taq polymerase (TaKaRa), 100 ng of templet DNA, 25 ml of 2 × GC buffer I (MgCl_2_^+^ plus), 0.4 mM dNTP, 0.5 μM of each primer, and double distilled H_2_O added to 50 μl. The reactions were carried out in a PTC-100 (MJ Research, Watertown, MA, USA) thermocycler using the following protocol: 94°C for 2 min, followed by 35 cycles of 94°C for 45 s, 58°C for 70 s and 72°C for 2 min, finally extended at 72°C for 10 min.

### Molecular cloning, DNA sequencing and sequences alignment

PCR products were separated on 1.2% agarose gels in Tris–acetic acid–EDTA buffer and expected fragments were purified from the gels using a Quick DNA extraction kit (Tiangen, Beijng, China). Subsequently, purified products were ligated into a PMD18–T Easy vector (TaKaRa, Dalian, China) and transformed into cells of *E. coli* strain DH5α according to Li et al. [75]. DNA sequencing was performed with three clones by Sino Geno Max, Beijing, China. Multiple sequence alignment of LMW glutenin nucleotide and protein sequences were completed by Bioedit 7.0.1.1.

### SNPs and InDels identification and secondary structure prediction

Identification of SNPs and InDels present in LMW glutenin genes were based on multiple alignments and performed using Bioedit 7.0. Prediction of secondary structure of deduced amino acid sequences was carried out by PSIPRED server (http://bioinf.cs.ucl.ac.uk/psipred/) [34].

### Phylogenetic analysis

MEGA 5 was used to construct a phylogenetic tree with the complete coding regions. Neighbor joining with Kimura two parameter correction methods and bootstrapping of 1,000 replicates were selected as working parameters [28,34,39,76,77].

### Expression of the cloned LMW-GS gene in *E. coli*

The gene cloned was re-amplified to remove the signal peptides by designing a new pair of primers CS-F (5’-GGG*CATATG*ATTTCACAGCAACAA-3’) and CS-R (5’-*CTCGAG*TCAGTAGACACCAACTCCGATG-3’), NdeI and XhoI sites (underlined) were incorporated into the 5’ ends of the CS-F and CS-R, respectively. After purification, the PCR products were ligated into the expression vector pET-30a (Novagen), and transformed into E.coli BL21 (DE3) plysS cells. And then we extract and separate the expressed protein from the E.coli, after that, we carried out them by SDS–PAGE according to Li et al. [75].

### mRNA extraction, cDNA synthesis and qRT-PCR

Developmental seeds from three spikes were combined together to extract total RNA from endosperm of CS and CS-n, and cDNA synthesis, qRT-PCR were according to Wang et al. [56]. The primers were: LMW-i-F: TGAAGACCTTCCTCGTCTTTG, LMW-i-R: CTGTGAAATTTGCGCAACG. Gene-specific primers were designed using Primer 5.0 and their specificities were checked by the melting curves of the RT-PCR products. Each qRT-PCR reaction was performed in 20 μl volumes containing 10 μl 2 × SYBR® Premix Ex Taq™ (TaKaRa), 2 μl 50-fold diluted cDNA, 0.4 μl of each gene-specific primer and 7.2 μl ddH2O. PCR conditions were as follows: 95°C for 3 min, 45 cycles of 15 s at 95°C, 57°C for 20s and 72°C for 20s. Three replicates were used for each sample. Reactions were conducted in a CFX96 Real-Time PCR Detection System (Bio-Rad). All data were analyzed with CFX Manager Software (Bio-Rad).

### Determination of *Glu-A3a* deletion in CS by STS-PCR marker

To identify the deletion of *Glu-A3a* in CS-n, we did the STS-PCR marker of the seven NILs, CS-1S^l^/1B, S genome addition line, CB037A, CS, CS-n. We used the marker name of glu-A3a to do PCR as what Wang et al. [24] did before. The primer sets are LA1F: AAACAGAATTATTAAAGCCGG, and SA1R: GGTTGTTGTTGTTGCAG CA. Their PCR cycling conditions were 94°C for 4 min, followed by 35 cycles of 94°C for 35 s, 55°C for 45 s, 72°C for 40 s, and a final extension at 72°C for 8 min.

### Development and validation of allele-specific PCR markers for *Glu-A3a*

To identify the *Glu-A3a* gene in different genotypes, based on the SNPs we detected in *Glu-A3a*, we designed the primer named *Glu-A3a* F: GCAAAGAAGGAAAAGAG GTGG, R: GGTTGTTGTTGTTGCTGCA as the primer to discriminate the gene *Glu-A3a* from others in CS and CS-n, this also validated in four RILs (CB037A and 1S^l^/1B), CS-1S^l^/1B, 1S^l^ genome addition line, 7 NILs of Aroona and 48 varieties. PCR cycling conditions were 94°C for 4 min, followed by 35 cycles of 94°C for 35 s, 60°C for 30 s, 72°C for 30 s, and a final extension at 72°C for 8 min.

## Additional files

Additional file 1: Figure S1. Plant, Spikelet and seeds morphology of CS and CS-n. A: Plant morphology of CS and CS-n. B1 and B2 are Spikelet morphology of CS and CS-n, respectively. C1 and C2 are the seeds of CS and CS-n, separately. Additional file 2: Figure S2. Kernel morphology of different developmental times of CS and CS-n. The seeds are 5, 8, 11, 14, 17, 20, 23, 26, 29 DPAs from CS and CS-n respectively. The characterization of them is similar. Additional file 3: Table S1. Comparison of data statistics about the two varieties (CS and CS-n). We statistics plant height, Ear length, stronger spikelet number, kernels per spike, grain weight per spike, Tiller number and thousand kernel weight (TKW) of CS, CS-n. Additional file 4: Figure S3. The comparison of the albumins, globulins and prolamins in CS and CS-n. 1 and 2 are the albumins of CS-n and CS, respectively. 3 is the globulins of CS-n, 4 is the globulins of CS. 5 and 6 are the prolamins of CS-n and CS, separately. The difference between them was marked by a black arrow. Additional file 5: Figure S4. Identification of *Glu-A3a* in LMW-GS deletion lines of CS and CS-n by MALDI-TOF–MS. The LMW-GS *Glu-A3a* is marked by a black arrow. Additional file 6: Figure S5. Agarose gel electrophoresis separation of amplified products from genomic DNA of CS. With the AS-PCR primer, a single band was cloned in CS. Lane1 and Lane2: PCR amplified products and Lane3: 1 kb DNA marker. Additional file 7: Figure S6. Sequence alignment of STS-PCR marker products of CS, *Glu-A3a* and its CDS. *Glu-A3a* is the full sequence, and the *Glu-A3a* CDS is the coding area. Marker is the band we cloned with the marker named *glu-A3a* from CS. Additional file 8: Table S2. Comparison of the mature protein sequences of different LMW-i glutenin subunits. The number and positions of cysteine in different LMW-i glutenin subunits are different and relative conservative. Additional file 9: Figure S7. qRT-PCR optimization design: double standard curve and dissolution curve of the gene *Glu-A3a.* The red standard curve represented *Glu-A3a* gene and the other blue standard curve represented the reference gene. The dissolution curves of different genes were indicated. Additional file 10: Table S3. Validation of the *Glu-A3a* allele-specific markers with different wheat varieties, a set of NILs and 4 RILs. The source and the allele that contained were listed in this file.

## Abbreviations

2D-PAGE: Two-dimensional gel electrophoresis (IEF × SDS-PAGE)
A-PAGE: Acid polyacrylamide gel electrophoresis
AS-PCR: Allele specific-polymerase chain reaction
BAC: Bacterial artificial chromosome
BIP: Binding protein
CAAS: Chinese Academy of Agricultural Sciences
CBB: Coomassie brilliant blue
DPA: Days post anthesis
GMP: Glutenin macropolymer
HMW-GS: High-molecular-weight glutenin subunits
InDels: Insertions/deletions
LC-MS/MS: Liquid chromatography-mass spectrometer/mass spectrometer
LMW-GS: Low-molecular-weight glutenin subunits
MALDI-TOF–MS: Matrix-assisted laser desorption/ionization time-of-flight mass spectrometry
MCC: Micro-core collections
NILs: Near-isogenetic lines
ORFs: Open reading frame
PDI: Protein disulfide isomerase
PCR: Polymerase chain reaction
qRT-PCR: Quantitative real-time polymerase chain reaction
RIL: Recombinant inbred line
RP-UPLC: Reversed-phase ultra performance liquid chromatography
Rmax: Extensograph maximum resistance
RWCN: Raw white Chinese noodle
SNPs: Single nucleotide polymorphisms
STS-PCR: Sequence-tagged site polymerase chain reaction
SDS-PAGE: Sodium dodecyl sulphate-polyacrylamide gel electrophoresis
ZSV: Zeleny sedimentation value
