# Supplementary figures and images for: Deletion of the low-molecular-weight glutenin subunit allele Glu-A3a of wheat (Triticum aestivum L.) significantly reduces dough strength and breadmaking quality
Source: BMC Plant Biol. 2014 Dec 19;14:367. doi: 10.1186/s12870-014-0367-3 (PMC4275963; doi:10.1186/s12870-014-0367-3)

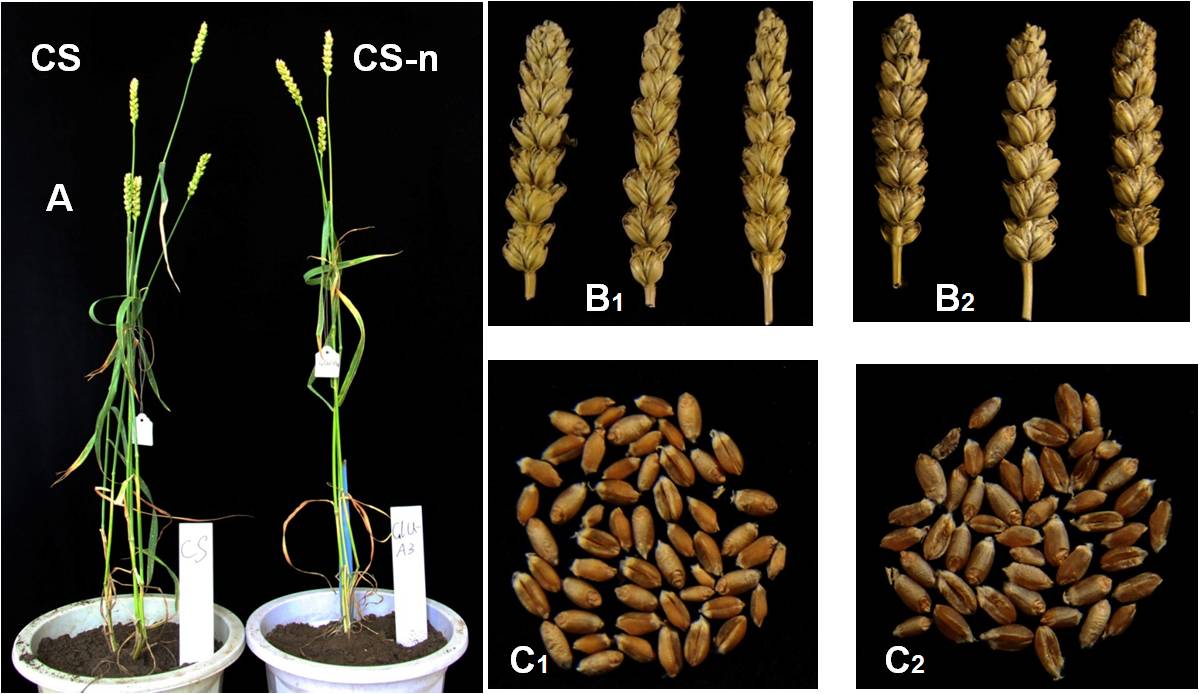

Supplement: Additional file 1: Figure S1. — Plant, Spikelet and seeds morphology of CS and CS-n. A: Plant morphology of CS and CS-n. B1 and B2 are Spikelet morphology of CS and CS-n, respectively. C1 and C2 are the seeds of CS and CS-n, separately. [file 12870_2014_367_MOESM1_ESM.jpeg]

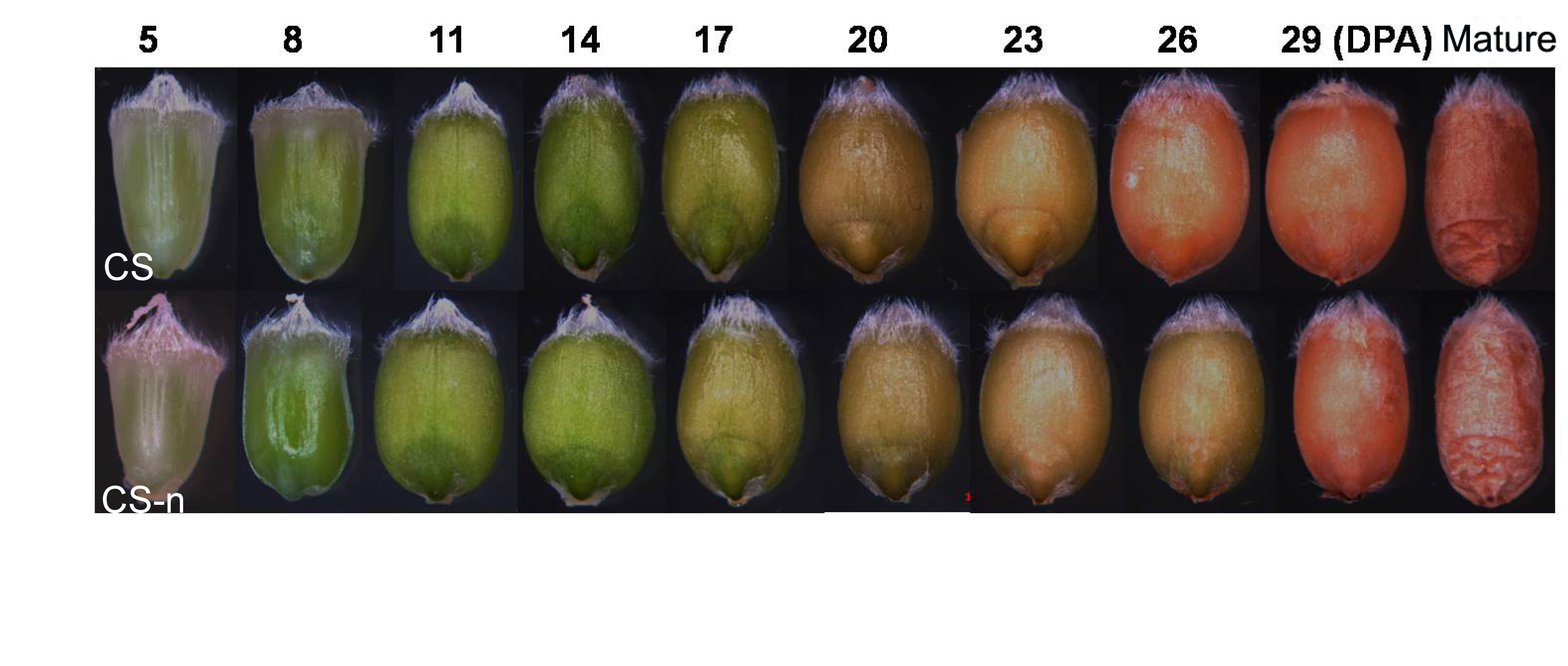

Supplement: Additional file 2: Figure S2. — Kernel morphology of different developmental times of CS and CS-n. The seeds are 5, 8, 11, 14, 17, 20, 23, 26, 29 DPAs from CS and CS-n respectively. The characterization of them is similar. [file 12870_2014_367_MOESM2_ESM.jpeg]

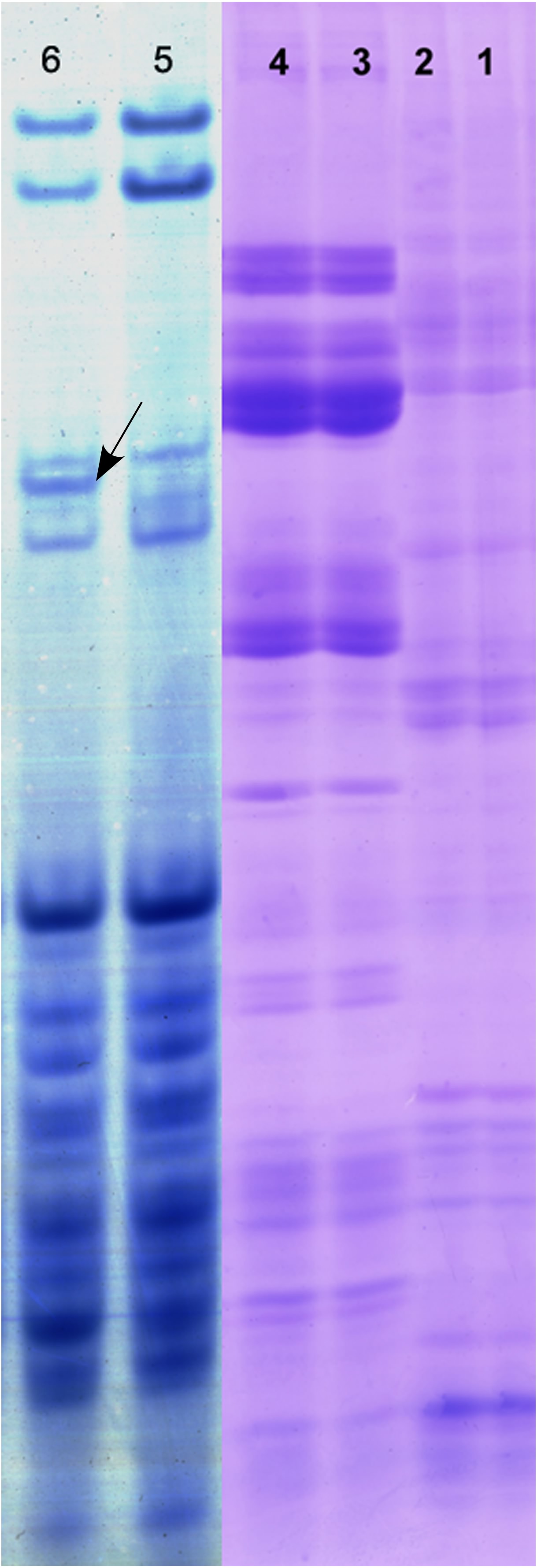

Supplement: Additional file 4: Figure S3. — The comparison of the albumins, globulins and prolamins in CS and CS-n. 1 and 2 are the albumins of CS-n and CS, respectively. 3 is the globulins of CS-n, 4 is the globulins of CS. 5 and 6 are the prolamins of CS-n and CS, separately. The difference between them was marked by a black arrow. [file 12870_2014_367_MOESM4_ESM.jpeg]

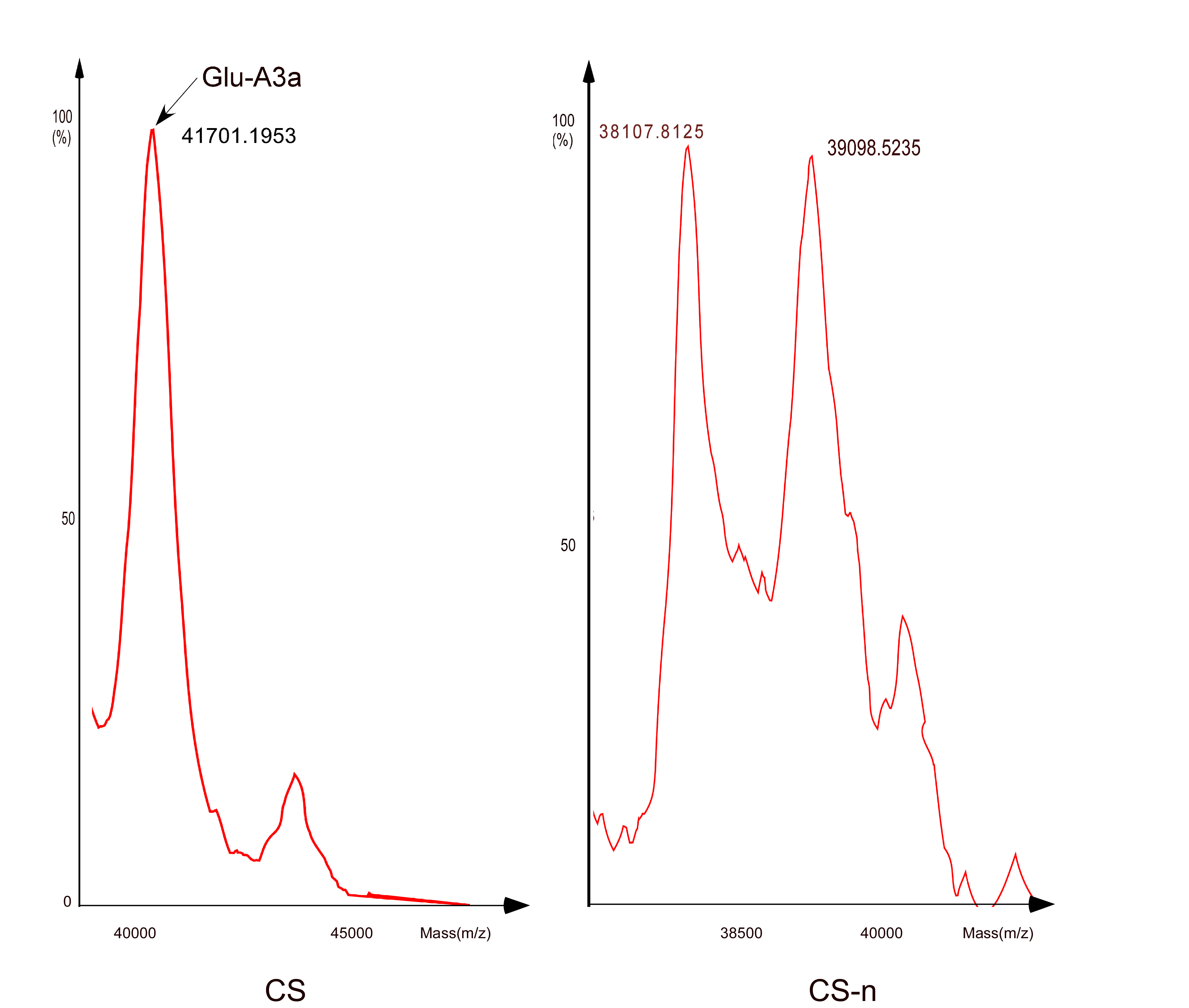

Supplement: Additional file 5: Figure S4. — Identification of Glu-A3a in LMW-GS deletion lines of CS and CS-n by MALDI-TOF–MS. The LMW-GS Glu-A3a is marked by a black arrow. [file 12870_2014_367_MOESM5_ESM.jpeg]

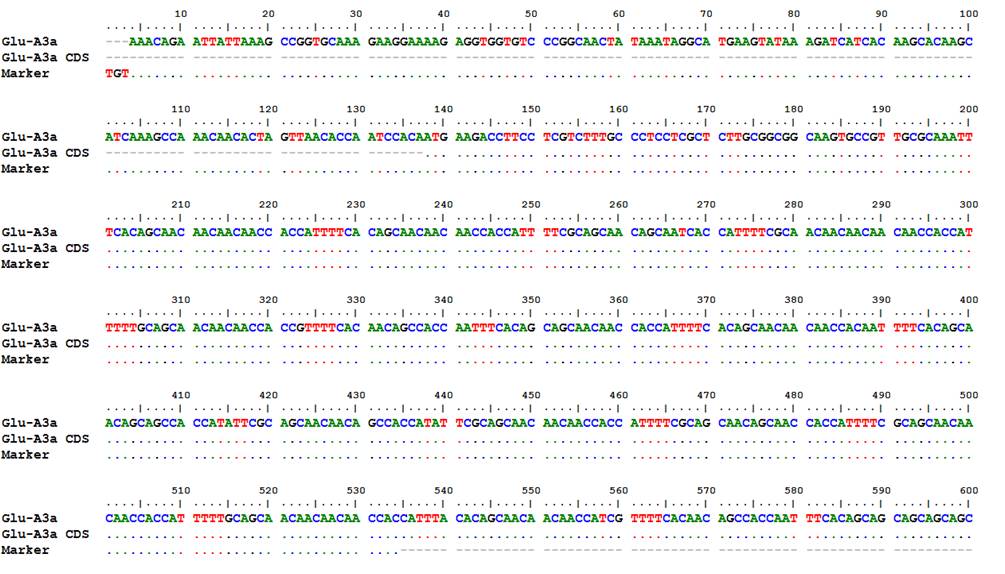

Supplement: Additional file 6: Figure S5. — Agarose gel electrophoresis separation of amplified products from genomic DNA of CS. With the AS-PCR primer, a single band was cloned in CS. Lane1 and Lane2: PCR amplified products and Lane3: 1 kb DNA marker. [file 12870_2014_367_MOESM6_ESM.jpeg]

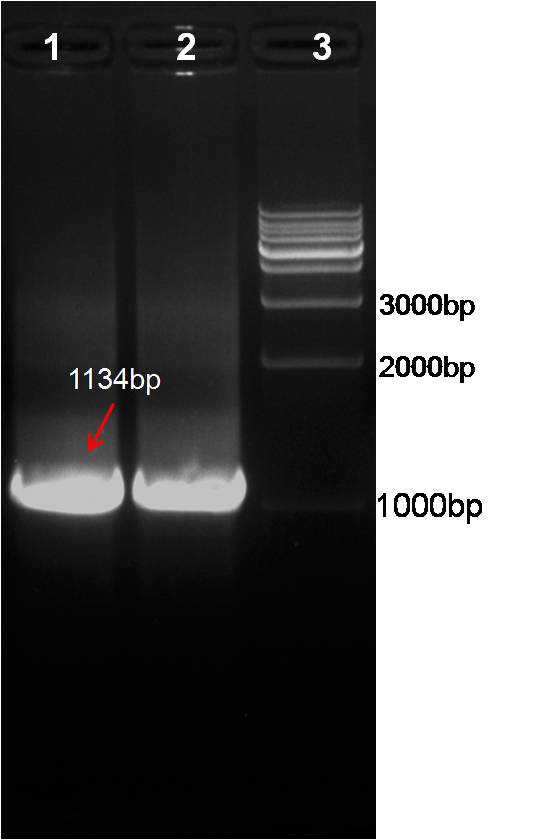

Supplement: Additional file 7: Figure S6. — Sequence alignment of STS-PCR marker products of CS, Glu-A3a and its CDS. Glu-A3a is the full sequence, and the Glu-A3a CDS is the coding area. Marker is the band we cloned with the marker named glu-A3a from CS. [file 12870_2014_367_MOESM7_ESM.jpeg]

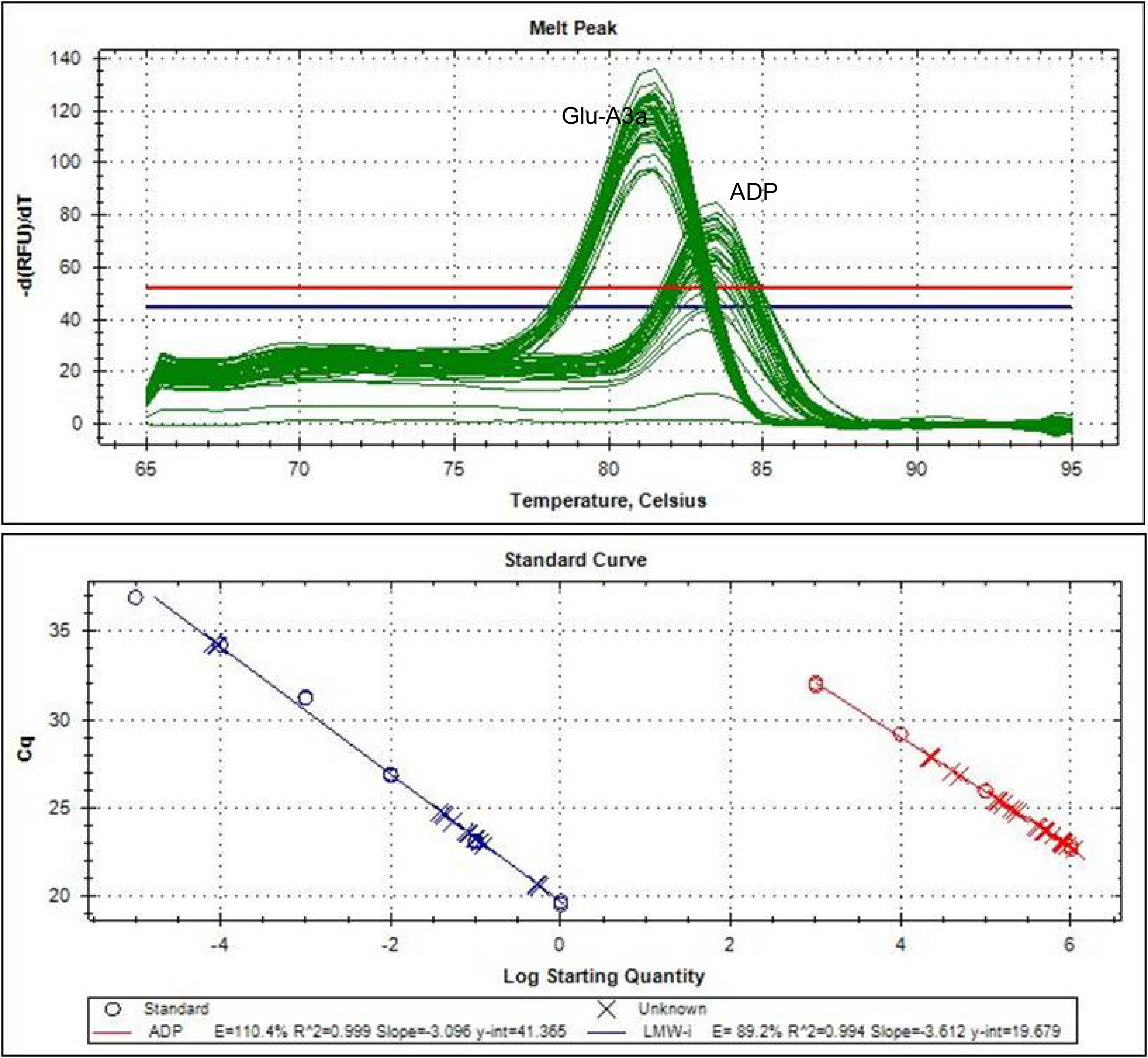

Supplement: Additional file 9: Figure S7. — qRT-PCR optimization design: double standard curve and dissolution curve of the gene Glu-A3a. The red standard curve represented Glu-A3a gene and the other blue standard curve represented the reference gene. The dissolution curves of different genes were indicated. [file 12870_2014_367_MOESM9_ESM.jpeg]
